# Supplementary material for: Quantifying the unquantifiable: why Hymenoptera, not Coleoptera, is the most speciose animal order
Source: BMC Ecol. 2018 Jul 12;18:21. doi: 10.1186/s12898-018-0176-x (PMC6042248; doi:10.1186/s12898-018-0176-x)
Supplement: Supplementary file 2 — Additional file 2. References for parasitoid lists in Tables S1–S4. [file 12898_2018_176_MOESM2_ESM.docx]

Additional References

Adams, A. S., and Six, D. L. 2008. Detection of host habitat by parasitoids using cues associated with mycangial fungi of the mountain pine beetle, *Dendroctonus ponderosae*. The Canadian Entomologist 140:124-127.

Baker, W.L. 1972. Eastern Forest Insects. United States Department of Agriculture Forest Service Miscellaneous Publication 1175:1-642.

Becker, G.C., and Benjamin, D. M. 1967. The biology of *Neodiprion nigroscutum* (Hymenoptera: Diprionidae) in Wisconsin. The Canadian Entomologist 99:146-159.

Benjamin, D.M. 1955. The biology and ecology of the red-headed pine sawfly. United States Department of Agriculture Forest Service Technical Bulletin 1118:1-57.

Berisford, C. W. 2011. Parasitoids of the southern pine beetle. Pages 129-139 in R. Coulson and K. Klepzig Southern Pine Beetle II. General Technical Report. SRS-140. Asheville, NC: US Department of Agriculture Forest Service, Southern Research Station.

Berisford, C. W., Kulman, H. M., and Pienkowski, R. L. 1970. Notes on the biologies of hymenopterous parasites of *Ips* spp. bark beetles in Virginia. The Canadian Entomologist 102:484-490.

Berlocher, S.H., 1984. A new North American species of Rhagoletis (Diptera: Tephritidae), with records of host plants of Cornus-infesting *Rhagoletis*. Journal of the Kansas Entomological Society 57:237-242.

Bird, R.D. 1929. Notes on the fir sawfly *Neodiprion abeitis* Harris. Annual Report of the Entomological Society of Ontario 1929:76-82.

Blatt, S. E., Knox, D. A., and Harmsen, R. 2000. Apple or cherry? Host selection quandary for the eastern tent caterpillar. Proceedings of the Entomological Society of Ontario 131:123-131.

Bobb, M. L. 1965. Insect parasite and predator studies in a declining sawfly population. Journal of Economic Entomology 58:925-926.

Bowers, W. W., Borden, J. H., and Raske, A. G. 1996. Bionomics of the four‐eyed spruce bark beetle, *Polygraphus rufipennis* (Kirby)(Col., Scolytidae) in Newfoundland II. Host colonization sequence. Journal of Applied Entomology 120:449-461.

Brown, M. W. 1984. Literature review of *Ooencyrtus kuvanae* [Hym.: Encyrtidae], an egg parasite of Lymantria dispar [Lep: Lymantriidae]. Entomophaga 29: 249-265.

Buckingham, G. R. 1975. The Parasites of Walnut Husk Flies (Diptera: Tephritidae: *Rhagoletis*) Including Comparative Studies on the Biology of *Biosteres Juglandis* Mues. (Hymenoptera: Braconidae) and on the Male Tergal Glands of the Braconidae (Hymenoptera). Doctoral dissertation, University of California.

Bugbee, R. E. 1967. Revision of Chalcid wasps of genus *Eurytoma* in America North of Mexico. Proceedings of the US National Museum 118: 433-552.

Bush, G. L. 1966. The taxonomy, cytology, and evolution of the genus *Rhagoletis* in North America (Diptera, Tephriditae). Bulletin of the Museum of Comparative Zoology 134:431–562.

Bushing, R. W. 1965. A synoptic list of the parasites of Scolytidae (Coleoptera) in North America north of Mexico. The Canadian Entomologist 97:449-492.

Carolina Blassioli Moraes, M., Laumann, R., Sujii, E. R., Pires, C., and Borges, M. 2005. Induced volatiles in soybean and pigeon pea plants artificially infested with the neotropical brown stink bug, *Euschistus heros*, and their effect on the egg parasitoid, Telenomus podisi. Entomologia Experimentalis et Applicata 115:227-237.

Choate, B. A., and Rieske, L. K. 2005. *Sympiesis fragariae* (Hymenoptera: Eulophidae) parasitizes *Malacosoma americanum* (Lepidoptera: Lasiocampidae) in Kentucky, USA: host and geographic records. Entomological news 116:183-185.

Coppel, H. C., and Benjamin, D. M. 1965. Bionomics of the nearctic pine-feeding diprionids. Annual review of entomology 10:69-96.

Costamagna, A. C., and Landis, D. A. 2004. Effect of food resources on adult *Glyptapanteles militaris* and *Meteorus communis* (Hymenoptera: Braconidae), parasitoids of *Pseudaletia unipuncta* (Lepidoptera: Noctuidae). Environmental entomology 33:128-137.

Coster, J. E., and Stein, C. 1977. Distribution of Some Predators and Parasites of the Southern Pine Beetle in Two Species of Pine. Environmental Entomology 6:689-694.

Cote III, W. A., and Allen, D. C. 1980. Biology of two-lined chestnut borer, *Agrilus bilineatus*, in Pennsylvania and New York. Annals of the Entomological Society of America 73:409-413.

Cushman, R. A. 1927. The parasites of the pine tip moth, *Rhyacionia frustrana* (Comstock). Journal of Agricultural Research 34:615-22.

Cushman, R. A. 1939. New Ichneumon-flies parasitic on the hemlock sawfly (*Neodiprion tsugae* Middleton). Journal of the Washington Academy of Sciences 29:391-402.

Cushman, R. A. 1940. A review of the parasitic wasps of the ichneumonid genus *Exenterus* Hartig. United States Department of Agriculture Miscellaneous Publication 354:1-15.

Dahlstein, D. L. 1967. Preliminary life tables for pine sawflies in the *Neodiprion fulviceps* complex (Hymenoptera: Diprionidae). Ecology 48:275-289.

Deyrup, M. A. 1975. The insect community of dead and dying Douglas-fir. I. The Hymenoptera. Seattle: Coniferous Forest Biome, University of Washington.

Doerksen, G. P., and Neunzig, H. H. 1976. Biology of Some Immature *Nephopterix* in the Eastern United States (Lepidoptera: Pyralidae: Phycitinae). Annals of the Entomological Society of America,69:423-431.

Drooz, A.T. 1985. Insects of Eastern Forests. United States Department of Agriculture Forest Service Miscellaneous Publication 1426:1-608.

Drooz, A. T., Wilkinson, R. C., and Fedde, V. H. 1977. Larval and cocoon parasites of three *Neodiprion* sawflies in Florida. Environmental Entomology 6:60-62.

Finlayson, T. 1961. Note on effects of some minerals on fecundity of *Aptesis basizona* (Grav.)(Hymenoptera: Ichneumonidae). The Canadian Entomologist 93:626-628.

Finlayson, T. 1963. Taxonomy of cocoons and puparia, and their contents, of Canadian parasites of some native Diprionidae (Hymenoptera). The Canadian Entomologist 95:475-507.

Fiske, W. F. 1903. A study of the parasites of the American tent caterpillar. New Hampshire College.

Forbes, A. A., Powell, T. H. Q., Stelinski, L. L., Smith, J. J., and Feder, J. L. 2009. Sequential sympatric speciation across trophic levels. Science 323:776-779.

Forbes, A.A., Satar, S., Hamerlinck, G., Nelson, A.E., Smith, J.J. 2012. DNA Barcodes and targeted sampling methods identify a new species and cryptic patterns of host specialization among North American *Coptera* (Hymenoptera: Diapriidae). The Annals of the Entomological Society of America 105:608-612.

Frank, J. H., and Foltz, J. L. 1997. Classical biological control of pest insects of trees in the southern United States: a review and recommendations (Vol. 96, No. 20). Forest Health Technology Enterprise Team.

Furniss, M. M. 1968. Notes on the biology and effectiveness of Karpinskiella paratomicobia parasitizing adults of *Dendtroctonus pseudotsugae*. Annals of the Entomological Society of America 61:1384-1389.

Furniss, R. L. and Dowden, P. B. 1941. Western hemlock sawfly, *Neodiprion tsugae* Middleton, and its parasites in Oregon. Journal of Economic Entomology 34:46-52.

Gahan, A. B. 1937. Two new chalcidoid egg parasites (Eulophidae and Mymaridae). Proceedings of the Entomological Society of Washington 39:266-269.

Gara, R. I., Werner, R. A., Whitmore, M. C., and Holsten, E. H. 1995. Arthropod associates of the spruce beetle *Dendroctonus rufipennis* (Kirby) (Col., Scolytidae) in spruce stands of south‐central and interior Alaska. Journal of Applied Entomology 119:585-590.

Gibson, G. A., and Floate, K. 2001. Species of *Trichomalopsis* (Hymenoptera: Pteromalidae) associated with filth flies (Diptera: Muscidae) in North America. The Canadian Entomologist 133:49-85.

Griffiths, K. J. 1960. Parasites of *Neodiprion pratti banksianae* Rohwer in northern Ontario. The Canadian Entomologist 92:653-658.

Hamerlinck, G., Hulbert, D., Hood, G.R., Smith, J.J., Forbes, A.A. 2016. Histories of host shifts and cospeciation among free-living parasitoids of *Rhagoletis* flies. Journal of Evolutionary Biology 29:1766-1779.

Hard, J. S. 1976. Natural control of hemlock sawfly, *Neodiprion tsugae* (Hymenoptera: Diprionidae), populations in southeast Alaska. The Canadian Entomologist 108:485-498.

Hervet, V. A. D. 2017. Host range and multitrophic interactions between the parasitoid *Cotesia vanessae* (Hymenoptera: Braconidae) and Noctuidae (Lepidoptera) hosts in North America. Doctoral dissertation, University of Lethbridge (Canada).

Hetrick, L. A. 1941. Life history studies of *Neodiprion americanum* (Leach). Journal of Economic Entomology 34:373-377.

Hetrick, L. A. 1959. Ecology of the pine sawfly, *Neodiprion excitans* (Rohwer) (Hymenoptera, Diprionidae). The Florida Entomologist 42:159-162.

Hopping, G. R. and Leech, H. B. 1936. Sawfly biologies. I. *Neodiprion tsugae* Middleton. The Canadian Entomologist 68:71-79.

Inayatullah, M., Shaw, S. R., and Quicke, D. L. J. 1998. The genus *Vipio* Latreille (Hymenoptera: Braconidae) of America north of Mexico. Journal of Natural History 32:117-148.

Jiménez Quiroz, E., Sánchez Escudero, J., Equihua Martínez, A., Montiel, M., Tuliaseso, J., and Valdez Carrasco, J. 2008. Distribución, abundancia y parasitismo de *Ooencyrtus kuvanae* (Howard) (Hymenoptera: Encyrtidae) parasitoide de los huevos de *Malacosoma incurvum* Hy. Edwards (Lepidoptera: Lasiocampidae) en Xochimilco, DF (No. TESIS.). Colegio de Postgraduados, Campus Montecillo, Campus Montecillo, Postgrado de Fitosanidad, Entomología y Acarología.

Juliano, S. A., and Borowicz, V. A. 1987. Parasitism of a frugivorous fly, *Rhagoletis cornivora*, by the wasp *Opius richmondi*: relationships to fruit and host density. Canadian journal of zoology 65:1326-1330.

Kapler, J. E., and Benjamin, D. M. 1960. The biology and ecology of the red-pine sawfly in Wisconsin. Forest Science 6(3).

Katovich, S. A., McCullough, D. G., and Haack, R. A. 1995. Yellowheaded spruce sawfly – It’s ecology and management. United States Department of Agriculture Forest Service General Technical Report NC-179:1-24.

Knerer, G., and Wilkinson, R. C. 1990. The biology of *Neodiprion pratti* (Dyar) (Hym., Diprionidae), a winter sawfly in West Florida. Journal of Applied Entomology 109:448-456.

Kraemer, M. E., and Coppel, H. C. 1978. The parasitoids of the European pine sawfly *Neodiprion sertifer* (Geoffroy) (Hymenoptera: Diprionidae), in Wisconsin, with keys to adults and larval remains. Wisconsin Academy of Sciences, Arts and Letters 66:91-112.

Krombein, K.V., Hurd, P.D., Smith, D.R. and Burks, B.D., 1979. Catalog of Hymenoptera in America north of Mexico. Smithsonian Institution Press. Washington, D.C.

Krugner, R., Daane, K. M., Lawson, A. B., and Yokota, G. Y. 2005. Biology of *Macrocentrus iridescens* (Hymenoptera: Braconidae): a parasitoid of the obliquebanded leafroller (Lepidoptera: Tortricidae). Environmental entomology 34:336-343.

Kulhavy, D., and Miller, M. C. 1989. Potential for biological control of *Dendroctonus* and *Ips* bark beetles. Stephen A. Austin State University Faculty Publications. 215.

Kulman, H. M. 1965. Natural control of the eastern tent caterpillar and notes on its status as a forest pest. Journal of Economic Entomology 58:66-70.

Langor, D. W. 1991. Arthropods and nematodes co-occurring with the eastern larch beetle, Dendroctonus simplex [Col.: Scolytidae], in Newfoundland. Entomophaga 36:303-313.

Langor, D. W., and Raske, A. G. 1987. Reproduction and development of the eastern larch beetle, *Dendroctonus simplex* LeConte (Coleoptera: Scolytidae), in Newfoundland. The Canadian Entomologist 119:985-992.

Langston, R. L. 1957. A synopsis of hymenopterous parasites of *Malacosoma* in California. University of California Publications in Entomology 14:1-50.

Lee, J. C., and Heimpel, G. E. 2005. Impact of flowering buckwheat on Lepidopteran cabbage pests and their parasitoids at two spatial scales. Biological Control 34:290-301.

Legault, S., Hébert, C., Blais, J., Berthiaume, R., Bauce, E., & Brodeur, J. 2012. Seasonal ecology and thermal constraints of *Telenomus* spp. (Hymenoptera: Scelionidae), egg parasitoids of the hemlock looper (Lepidoptera: Geometridae). Environmental entomology 41:1290-1301.

Leius, K. 1967. Influence of wild flowers on parasitism of tent caterpillar and codling moth. The Canadian Entomologist 99:444-446.

Linit, M. J., and Stephen, F. M. 1983. Parasite and predator component of within-tree southern pine beetle (Coleoptera: Scolytidae) mortality. The Canadian Entomologist 115: 679-688.

Liu, C. L. 1926. On Some Factors of Natural Control of the Eastern Tent Caterpillar (*Malacosoma americana* Harris): With Notes on the Biology of the Host. Cornell University.

Lyons, L. A. 1962. The effect of aggregation on egg and larval survival in *Neodiprion swainei* Midd. (Hymenoptera: Diprionidae). The Canadian Entomologist 94:49-58.

Lyons, L. A. 1964. The European pine sawfly, *Neodiprion sertifer* (Geoff.) (Hymenoptera: Diprionidae). A review with emphasis of studies in Ontario. Proceedings of the Entomological Society of Ontario 94:5-37.

Marsh, P. M. 1968. The Nearctic Doryctinae, VII. The genus *Doryctes* Haliday (Hymenoptera: Braconidae). Transactions of the American Entomological Society 94:379-405.

Mason, W. R. M. 1978. A synopsis of the Nearctic Braconini, with revisions of Nearctic species of *Coeloides* and *Myosoma* (Hymenoptera: Braconidae). The Canadian Entomologist 110:721-765.

Mason, W. R. M. 1979. A new *Rogas* (Hymenoptera: Braconidae) parasite of tent caterpillars (*Malacosoma* spp. Lepidoptera: Lasiocampidae) in Canada. Canadian Entomologist 111: 783-786.

Mathews, P. L., and Stephen, F. M. 1997. Effect of artificial diet on longevity of adult parasitoids of *Dendroctonus frontalis* (Coleoptera: Scolytidae). Environmental entomology 26:961-965.

McGovern, W. L., Cross, W. H., and Mitchell, H. C. 1974. *Eupelmus cyaniceps* (Hymenoptera: Eupelmidae) a hyperparasite. Journal of the Georgia Entomological Society 9:68-69.

McGregor, M. D., and Sandin, L. O. 1968. Observations on the pinyon pine sawfly, *Neodiprion edulicolus*, in eastern Nevada (Hymenoptera: Diprionidae). The Canadian Entomologist 100:51-57.

Minnoch, M. W., and Parker, D. L. 1971. Life History of a Looper, *Lambdina punctata*, in Utah (Lepidoptera: Geometridae). Annals of the Entomological Society of America 64:386-389.

Morris, C. L., and Schroeder, W. J. 1966. Pine cone mortality. Va Forests 21:18, 20.

Moser, J. C., Thatcher, R. C., and Pickard, L. S. 1971. Relative Abundance of Southern Pine Beetle 1 Associates in East Texas. Annals of the Entomological Society of America 64:72-77.

Muesebeck, C. F. W. 1980. The Nearctic parasitic wasps of the genera *Psilus* Panzer and *Coptera* Say (Hymenoptera, Proctotrupoidea, Diapriidae). United States Department of Agriculture Technical Bulletin 1617:1-71.

Newcomer, E. J. 1936. Effect of cold storage on eggs and young larvae of codling moth. Journal of Economic Entomology 29:1123-1125.

Noyes, J. S. 2017. Universal Chalcidoidea Database. World Wide Web electronic publication. <http://www.nhm.ac.uk/chalcidoids>

Overgaard, N. A. 1968. Insects associated with the southern pine beetle in Texas, Louisiana, and Mississippi. Journal of Economic Entomology 61: 1197-1201.

Packard, A. S. 1881. Insects injurious to forest and shade trees. United States Entomological Commission Bulletin 7: 1-275.

Parry, D. 1995. Larval and pupal parasitism of the forest tent caterpillar, *Malacosoma disstria* Hübner (Lepidoptera: Lasiocampidae), in Alberta, Canada. The Canadian Entomologist 127:877-893.

Peck, O. 1963. A catalogue of the nearctic Chalcidoidea (Insecta: Hymenoptera). The Memoirs of the Entomological Society of Canada 95(S30):5-1092.

Porter, B. A. 1917. The host of *Ablerus clisiocampae* Ash. Entomological News 28:186.

Porter, B. A., and C. H. Alden. 1921. *Anaphoidea conotracheli* Girault (Hym.), and egg parasite of the apple maggot. Proceedings of the Entomological Society of Washington 23:62–63.

Price, P. W. 1970. Characteristic permitting coexistence among parasitoids of a sawfly in Quebec. Ecology 51:445-454.

Price, P. W., and Tripp, H. A. 1972. Activity patterns of parasitoids on the Swaine jack pine sawfly, *Neodiprion swainei* (Hymenoptera: Diprionidae), and parasitoid impact on the host. The Canadian Entomologist 104:1003-1016.

Raizenne, H. 1957. Forest sawflies of southern Ontario and their parasites. Canada Department of Agriculture Publication 1009:1-44.

Rauf, A., and Benjamin, D. M. 1980. The biology of the white pine sawfly, *Neodiprion pinetum* (Hymenoptera: Diprionidae) in Wisconsin. The Great Lakes Entomologist 13:219-224.

Riley, M. A., and Goyer, R. A. 1986. Impact of Beneficial Insects on *Ips* spp. (Coleoptera: Scolytidue) Bark Beetles in Felled Loblolly and Slash Pines in Louisiana. Environmental Entomology 15:1220-1224.

Saffer, B. 1982. Systematic revision of the genus *Cenocoelius* (Hymenoptera, Braconidae) in North America including Mexico. Polish Journal of Entomology 52:73-167.

Schaffner Jr, J. V. 1934. Introduced parasites of the brown-tail and gipsy moths reared from native hosts. Annals of the Entomological Society of America 27:585-592.

Shaw, M.R. 2002. Host ranges of *Aleiodes* species (Hymenoptera: Braconidae), and an evolutionary hypothesis. Pages 321-327 *in*: G. Melika and C. Thuróczy (eds.), Parasitic wasps: Evolution, Systematics, Biodiversity and Biological Control. Agroinforum, Budapest.

Shaw, S. R. 2006. *Aleiodes* wasps of eastern forests: a guide to parasitoids and associated mummified caterpillars. US Department of Agriculture, Forest Service.

Smith, D. R. 1996. Aulacidae (Hymenoptera) in the mid-Atlantic states, with a key to species of eastern North America. Proceedings of the Entomological Society of Washington 98:274-291.

Stacey, L., Roe, R., and Williams, K. 1975. Mortality of eggs and pharate larvae of the eastern tent caterpillar, *Malacosoma americana* (F.) (Lepidoptera: Lasiocampidae). Journal of the Kansas Entomological Society 48:521–523

Stehr, F. W., and E. F. Cook. 1968. Revision of the genus *Malacosoma* Hübner in North America (Lepidoptera: Lasiocampidae): systematics, biology, immatures, and parasites. Bulletin (United States National Museum) 276:1-321

Stelzer, M. J. 1968. The Great Basin tent caterpillar in New Mexico: life history, parasites, disease and defoliation. U.S. Forest Service, Rocky Mountain Forest Experimental Station Paper 39.

Stevenson, R. E. 1967. Notes of the Biology of the Engelmann Spruce Weevil, *Pissodes engelmanni* (Curculionidae: Coleoptera) and its Parasites and Predators. The Canadian Entomologist 99:201-213.

Struble, G. R. 1957. Biology and control of the white-fir sawfly. Forest Science 3:306-313.

Sullivan, B. T., Pettersson, E. M., Seltmann, K. C., and Berisford, C. W. 2000. Attraction of the bark beetle parasitoid *Roptrocerus xylophagorum* (Hymenoptera: Pteromalidae) to host-associated olfactory cues. Environmental Entomology 29:1138-1151.

Townes, H., and Townes, M. 1960. Ichneumon-Flies of America North of Mexico Pt. 2: Subfamilies Ephialtinae, Xoridinae, and Acaenitinae. Memoirs of the American Entomological Institute 12:1-537.

Treherne, R. C. 1921. A further review of applied entomology in British Columbia. Journal of the Entomological Society of British Columbia 135-146.

Vanlaerhoven, S. L., Stephen, F. M., and Browne, L. E. 2005. Adult parasitoids of the southern pine beetle, *Dendroctonus frontalis* Zimmermann (Coleoptera: Scolytidae), feed on artificial diet on pine boles, pine canopy foliage and understory hardwood foliage. Biocontrol science and technology 15:243-254.

Walkley, L. M. 1954. A new cryptine genus of economic interest (Hymenoptera: Ichneumonidae). Journal of the Washington Academy of Sciences 44:219-220.

Wegensteiner, R., Wermelinger, B., and Herrmann, M. 2015. Natural enemies of bark beetles: predators, parasitoids, pathogens, and nematodes. Pages 247-304 i*n* F. E. Vega & R. W. Hofstetter (eds.), Bark Beetles. Biology and Ecology of Native and Invasive Species Elsevier: Amsterdam

Wellington, W. G. 1965. Some maternal influences on progeny quality in the western tent caterpillar, *Malacosoma pluviale* (Dyar). The Canadian Entomologist 97:1-14.

Wetzel, B. W., Kulman, H. M., and Witter, J. A. 1973. Effects of Cold Temperatures on Hatching of the Forest Test Caterpillar, *Malacosoma disstria* (Lepidoptera: Lasiocampidae). The Canadian Entomologist, 105:1145-1149.

Wharton, R.A., 1997. Generic relationships of opiine Braconidae (Hymenoptera) parasitic on fruit-infesting Tephritidae (Diptera). Journal of the Washington Academy of Sciences. 68:147–167.

Wharton, R. A. and Marsh, P. M. 1978. New World Opiinae (Hymenoptera: Braconidae) parasitic on Tephritidae (Diptera). Journal of the Washington Academy of Sciences 68:147-167.

Wharton, R., Ward, L., and Miko, I. 2012. New neotropical species of Opiinae (Hymenoptera, Braconidae) reared from fruit-infesting and leafmining Tephritidae (Diptera) with comments on the *Diachasmimorpha mexicana* species group and the genera *Lorenzopius* and *Tubiformopius*. ZooKeys 243:27-82.

Wharton, R. A., and Yoder, M. J. Parasitoids of Fruit-Infesting Tephritidae. http://paroffit.org. Accessed on Wed Dec 06 12:22:40 -0600 2017.

Wilkinson, R. C. 1971. *Neodiprion excitans* (Hymenoptera: Diprionidae) on sand pine in Florida. The Florida Entomologist 54:343-344.

Wilkinson, R. C., Becker, G. C., and Benajmin, D. M. 1966. The biology of *Neodiprion rugifrons* (Hymenoptera: Diprionidae), a sawfly infesting jack pine in Wisconsin. Annals of the Entomological Society of America 59:786-792.

Wilkinson, R. C., and Chellman, C. W. 1978. A new sawfly on sand pine in West Florida. Florida entomologist 61:26.

Wilkinson, R. C., and Drooz, A. T. 1979. Oviposition, Fecundity, and Parasites of *Neodiprion excitans* from Belize, CA. Environmental Entomology 8:501-505.

Williams, L. T. 1916. Notes on the egg-parasites of the apple tree tent-caterpillar (*Malacosoma americanum*). Psyche 23:148-153.

Witter, J. A., and Kulman, H. M. 1972. Review of the parasites and predators of tent caterpillars (*Malacosoma* spp.) in North America. Agricultural Experiment Station University of Minnesota. Techincal Bulletin 289.

Witter, J.A. and Kulman, H.M., 1979. The parasite complex of the forest tent caterpillar in northern Minnesota. Environmental Entomology, 8:723-731.

Yee, W. L., Goughnour, R. B., Hood, G. R., Forbes, A. A., and Feder, J. L. 2015. Chilling and host plant/site-associated eclosion times of Western cherry fruit fly (Diptera: Tephritidae) and a host-specific parasitoid. Environmental Entomology 44:1029-1042.
